# Novel Antimicrobial Titanium Dioxide Nanotubes Obtained through a Combination of Atomic Layer Deposition and Electrospinning Technologies

**DOI:** 10.3390/nano8020128

**Published:** 2018-02-24

**Authors:** Carol López de Dicastillo, Cristian Patiño, María Jose Galotto, Juan Luis Palma, Daniela Alburquenque, Juan Escrig

**Affiliations:** 1 Food Packaging Laboratory (Laben-Chile), Department of Science and Food Technology, Faculty of Technology, Universidad de Santiago de Chile (USACH), Obispo Umaña 050, 9170201 Santiago, Chile; cristian.patino@usach.cl (C.P.); maria.galotto@usach.cl (M.J.G.); 2 Center for the Development of Nanoscience and Nanotechnology (CEDENNA), 9170124 Santiago, Chile; juanluispalma@gmail.com (J.L.P.); daniela.alburquenquem@usach.cl (D.A.); juan.escrig@usach.cl (J.E.); 3 Department of Basic Sciences, Engineering Faculty, CIDES, Universidad Central de Chile, Santa Isabel 1186, 8330601 Santiago, Chile; 4 Department of Physics, Universidad de Santiago de Chile (USACH), Av. Ecuador 3493, 9170124 Santiago, Chile

**Keywords:** nanotechnology, atomic layer deposition, electrospinning, titanium dioxide nanotubes, antimicrobial

## Abstract

The search for new antimicrobial substances has increased in recent years. Antimicrobial nanostructures are one of the most promising alternatives. In this work, titanium dioxide nanotubes were obtained by an atomic layer deposition (ALD) process over electrospun polyvinyl alcohol nanofibers (PVN) at different temperatures with the purpose of obtaining antimicrobial nanostructures with a high specific area. Electrospinning and ALD parameters were studied in order to obtain PVN with smallest diameter and highest deposition rate, respectively. Chamber temperature was a key factor during ALD process and an appropriate titanium dioxide deposition performance was achieved at 200 °C. Subsequently, thermal and morphological analysis by SEM and TEM microscopies revealed hollow nanotubes were obtained after calcination process at 600 °C. This temperature allowed complete polymer removal and influenced the resulting anatase crystallographic structure of titanium dioxide that positively affected their antimicrobial activities. X-ray analysis confirmed the change of titanium dioxide crystallographic structure from amorphous phase of deposited PVN to anatase crystalline structure of nanotubes. These new nanostructures with very large surface areas resulted in interesting antimicrobial properties against Gram-positive and Gram-negative bacteria. Titanium dioxide nanotubes presented the highest activity against *Escherichia coli* with 5 log cycles reduction at 200 μg/mL concentration.

## 1. Introduction

In the recent past, foodborne illnesses and nosocomial infections occurred during hospitalization have been identified as two major problems that have been producing many economic and human losses. Nosocomial infections represent around 1.4 million infections every year [1,2]. In addition, several studies have evidenced that the widespread use of antibiotics has led to bacteria resistance to nearly all antibiotics. These problems have induced the need and interest of the scientific society in the search for powerful antimicrobial substances. Recently, nanotechnology is considered a useful technology in treating bacterial infections since the mode of action of nanoparticles is in direct contact with the bacteria cell wall without the need to penetrate them, being less prone to promote resistance in bacteria than antibiotics. The development of novel and efficient nanotechnological-based antimicrobial agents is among one of the priority areas in biomedical research [3,4,5]. Several nanoparticles (NPs) have demonstrated broad-spectrum antibacterial properties against both Gram-positive and Gram-negative bacteria, mainly silver NPs, zinc oxide NPs, carbon nanotubes and iron oxide NPs [6,7,8]. The control of NPs morphology and structure is an important factor because antimicrobial performance is highly influenced by the morphology, shape and size of the particles [9,10]. The increase in surface area or the design of an appropriate sized and shaped nanoparticle with desirable surface properties can lead to an improvement of bactericidal activity. Some works have already evidenced that silver NPs interacts with bacteria, fungi and viruses in a shape-dependent manner [11,12,13,14,15]. Consequently, the development of new nanostructures with different morphologies has recently attracted wide attention. In this work, electrospinning (EP) and atomic layer deposition (ALD) technologies were combined with the objective to develop novel antimicrobial nanotubes with well-defined nanoscale walls composed of titanium dioxide.

EP is regarded as one of the most widespread techniques offering rapid, inexpensive, simple, effective and relatively large-scale fabrication for fibrous structures. An electrical potential is applied between a droplet of a polymer solution held at the end of a capillary tube and grounded target and when the applied electric field overcomes the surface tension of the droplet, a charged jet of polymer solution is ejected and is controlled by the electric field [16,17]. On the other hand, ALD has been recognized as a key technology for the surface modification and the fabrication of nanostructures materials because it is the only applicable technique for the deposition of conformal and homogenous thin films [18]. In addition, this technique allows to deposit thin films in structures with complex geometries allowing to synthesize high aspect ratio nanostructures without shadowing effects [19]. ALD is based on a reaction between precursor materials which are separated into successive surface reactions separated by a purge step to remove the unreacted precursors and the by-product. The deposition of metal oxides involves the reaction between a metal complex (e.g., metal halide, metal alkoxide, etc.) and an oxygen source (water, ozone or oxygen peroxide). Under hydrolytic conditions, the formation of M-O-M (metal-oxo dimers) bonds is separated into the two successive reactions: (i) a hydrolysis step during the water pulse forming -OH groups and metal-oxo bonds and (ii) a condensation step in which the resulting hydroxyl groups react with the metal oxide precursors supplied by the next pulse.

Other works have already reported this combination of techniques as an attractive strategy to obtain new nanostructures with photonic, electronic, catalytic, nanofluidic and drug delivery applications [20,21,22]. Different metal oxide nanotubes have been developed using different methodologies, precursors and fibers. Specifically, titanium dioxide nanotubes have been obtained using ALD and EP techniques but these works were mainly focused on their characterization [23,24,25]. However, no development has been carried out with antimicrobial purposes.

On the other hand, titanium dioxide (TiO_2_) is a thermally stable and biocompatible chemical compound with high photocatalytic activity and has presented good results against bacterial contamination [26,27]. TiO_2_ has also become the preferred photocatalyst for a variety of reasons, which include its low cost, chemical stability, non-toxicity and effectiveness under near-ultraviolet light (300–400 nm). It is one of the most applied nanomaterials and is widely used as additive in food and non-food applications [28,29,30]. Other TiO_2_ nanostructures have been already developed with antimicrobial goals. TiO_2_ nanotubes obtained through a chlorine-based electrochemical anodization method when exposed to UV light during 24 h presented approximately one and three log reductions against *E. coli* and *S. aureus*, respectively, with starting bacterial working solutions at 10^3^ cfu/mL. Recently, Jian et al. have developed TiO_2_ and silver loaded-TiO_2_ antibacterial agents through sol-assay method with minimum inhibition concentration values around 1.6 mg/mL [31,32].

## 2. Experimental Section

### 2.1. Materials

#### 2.1.1. Polymers, Chemicals and Microorganisms

Gohsenol type AH-17 polyvinyl alcohol (PV) (saponification degree 97–98.5% and viscosity 25–30 mPa·s) was obtained from The Nippon Synthetic Chemical Co. (Osaka, Japan). Tetrakis (dimethylamide) titanium (TDMAT) (99.99% trace metals basis) and titanium dioxide nanoparticles (TiO_2_ NPs, 21 nm particle size) were obtained from Sigma Aldrich (Santiago, Chile).

*Escherichia coli* ATCC 25922, *Listeria innocua* ATCC 33090 and *Staphylococcus aureus* ATCC 25923 were chosen as Gram-negative and Gram-positive bacteria models. Bacterial strains were obtained from Biotechnology and Applied Microbiology Laboratory (LAMAP) (Santiago, Chile) and stored in glycerol 30% at −80 °C until needed. For experimental use, the stock cultures were maintained in tryptone soy agar slants at 4 °C and transferred monthly. Prior to each experiment, a loopful of each strain was transferred to 5 mL of tryptone soy agar and incubated at 37 °C for 16 h to obtain fresh early-stationary phase cells.

UVA lamp: 15W Philips UV-A (model Actinic BL TL TL-D 15W/10 1SL/25) (Amsterdam, The Netherland) light bulb was located 25 cm above the samples.

#### 2.1.2. Electrospun PV Nanofibers

Poly (vinyl alcohol) nanofibers were obtained with a polymeric solution at 8% (*w*/*w*) using an electrospinning system (Spraybase^®^power Supply Unit, Maynooth, Ireland). 1.6 g poly (vinyl alcohol) (PV) was added to 20 mL of distilled water and stirred at 90 °C until polymer was dissolved. Solution was transferred to 5 mL plastic syringes and connected through a PTFE tube to a 16-gauge blunt (1.6 mm diameter) stainless steel needle charged by a high voltage power supply with a range of 0–20 kV, using 1.5 mL/h as flow rate and 10 cm was the distance between the needle and the collector. Electrospun PV nanofibers were named “PVN.” The parameters of electrospinning system, such as distance (height between tip of the needle and collector plate), diameter of needle and flow rate, were previously studied in order to determine their effect on the nanofiber diameter (Supplementary Information, Section S1.1).

#### 2.1.3. Atomic Layer Deposition Process (ALD)

Coated electrospun PV nanofibers were obtained through the deposition of thin TiO_2_ layers using a Savannah S100 ALD equipment from Ultratech (San Jose, CA, USA) at different temperatures and purge times. During this process, the precursors Tetrakis (dimethylamide) titanium (TDMAT) and ultra-pure water were used as Ti and oxide sources, respectively. During cycling, the TDMAT and H_2_O were alternately introduced into the ALD chamber with pulse time of 0.1 and 0.015 s, respectively. N_2_ was used as a carrier gas at a flow rate of 20 sccm. The temperatures of TDMAT and H_2_O were set to 75 °C and room temperature, respectively. Three different temperatures in the ALD chamber were applied in order to study the influence of temperature in the process: (a) 500 cycles at 150 °C with a purge time of 20 s; (b) 500 cycles at 200 °C with a purge time of 10 s; and (c) 250/250 cycles at 150/250 °C with a purge time of 20/5 s and coated PV nanofibers obtained for each temperature condition were named PVN_150, PVN_200 and PVN_150/250, respectively. Since inert gas purge time following the H_2_O pulse is a critical parameter to obtain a linear growth regime [33], purge time used for every temperature were according to the provider’s recipe Cambridge NanoTech (Supplementary Information, Section S1.2).

#### 2.1.4. Polymer Template Removal

Two methodologies were carried out to remove PV polymeric nanofiber in order to obtain hollow titanium dioxide nanotubes (TDN): Method (A) by washing: based on the hydrosoluble nature of PV polymer. Deposited samples were sonicated into water, previously warmed at 90 °C, during 30 min. Washing water was removed and a second washing was done under a vigorous stirring at 90 °C during 3 h (sample TDN_A); and Method (B) by heating: based on polymeric thermal decomposition. Two temperatures were studied in order to observe the effect of temperature on polymer removal and TiO_2_ crystallization state. Coated nanofibers were thermal treated at 400 °C (sample TDN_B400) and 600 °C (sample TDN_B600), both during 2 h.

### 2.2. Characterization of Nanostructures: Nanofibers and Nanotubes

#### 2.2.1. Electron Microscopy (SEM and TEM)

The morphologies of PVN before and after ALD process and TiO_2_ nanotubes (TDN) obtained after the polymer template removal were studied by scanning electron microscopy (Zeiss EVO MA10 SEM, Oberkochen, Germany) at 20 kV and transmission electron microscopy (Hitachi HT7700 high resolution TEM, Chiyoda, Tokyo, Japan) at 100 kV. Nanostructures images were recorded at different magnifications.

#### 2.2.2. X-ray Diffraction (XRD)

XRD patterns were measured using a Siemens Diffractometer D5000 (30 mA and 40 kV) (Munich, Germany) using CuKa (λ = 1.54 Å) radiation at room temperature. All scans were performed in a 2θ range 2–80° at 0.02°/s. Debye-Scherrer’s equation was used to calculate the crystallites size (nm).

#### 2.2.3. Fourier Transform Infrared Spectroscopy (FTIR)

The presence of specific functional groups in the developed materials was analyzed by spectrometer equipment Bruker Alpha (Ettlingen, Karlsruhe, Germany) with accessory to make transmission spectra. Pellets with sample and potassium bromide (KBr) were prepared by pressure and the spectra were obtained in a range from 4000 to 400 cm^−1^, with a resolution of 2 cm^−1^ and 64 scans.

#### 2.2.4. Thermal Properties

Thermogravimetric analyses (TGA) were carried out using a Mettler Toledo Gas Controller GC20 Stare System TGA/DCS (Schwerzenbach, Switzerland). Samples (ca. 7 mg) were heated from 20 to 800 °C at 10 °C min^−1^ under nitrogen atmosphere (flow rate 50 mL min^−1^). PV polymer was also analyzed in order to study the effect of electrospinning process on polymer degradation.

### 2.3. Antimicrobial Activity of TiO_2_ Hollow Nanotubes

The antimicrobial activity of titanium dioxide nanotubes (TDN) developed in this work was tested under dynamic contact conditions against *Listeria innocua* and *Staphylococcus aureus*, as Gram-positive bacteria and *Escherichia coli*, as Gram-negative bacteria, following the International Normative ASTM E2149-10 with some modifications. TDN solutions at 100, 150, 200 and 400 µg/mL were made up in sterile buffer to study the antimicrobial activity. Commercial TiO_2_ NPs were also analyzed in order to compare antimicrobial activities. Cell cultures of each microorganism in stationary phase measured at 600 nm were diluted in TSB and incubated at 37 °C until reach the exponential phase corresponding with a bacterial concentration of 10^8^ CFU/mL. Triple 1/10 dilution was done, obtaining a final concentration of 10^5^ CFU/mL bacteria. Compounds were put in contact with bacterial solution and irradiated for 3 h using UVA lamp. Immediately after this irradiation period, 10 µL of every experimental solution was grown in a Petri dish at 37 °C by 24 h. Bacterial solutions with and without UVA irradiation were used as controls. Antimicrobial activity was expressed as log cycles reduction through Equation (1):

Log (cycles) Reduction = Log_10_ (control) − Log_10_ (experimental solution) (1)

### 2.4. Statistical Analysis

An analysis of variance (ANOVA) of microbiological studies was carried out. Fisher test (α = 0.05) was used to calculate the minimum significant difference among samples. All calculations were carried out with XLSTAT Pro software 2015 (Addinsoft, Paris, France).

## 3. Results and Discussion

### 3.1. Morphological Results of Nanofibers and Nanotubes

**Figure 1:**
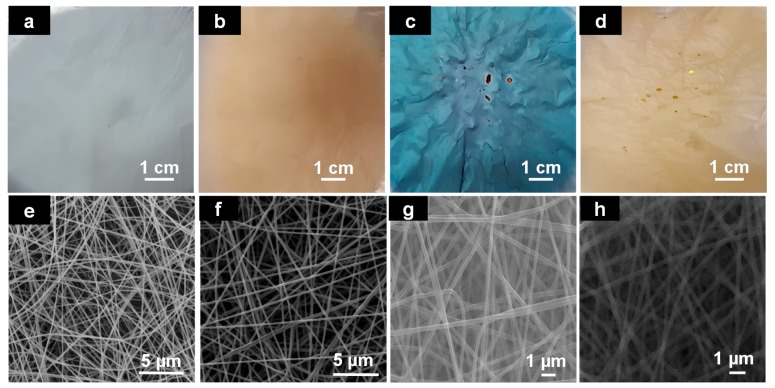
Photographs of electrospun fibers (PVN) (**a**) and deposited samples with TiO_2_ at different temperatures: (**b**) PVN_150, (**c**) PVN_200; (**d**) PVN_150/250; and SEM images of: (**e**) PVN, (**f**) PVN_150, (**g**) PVN_200, (**h**) PVN_150/250.

As Figure 1a,e show, electrospun PVN were successfully obtained and presented a diameter average of 164.5 ± 24.7 nm and exhibited smooth surfaces. These fibers were then coated with TiO_2_ by using ALD at different temperatures and as Figure 1b–d show, resulting samples “PVN_150, PVN_200 and PVN_150/250” presented different colors. Additionally, SEM images (Figure 1e–h) demonstrated that the mold structure of the PV nanofibers was maintained and deposited nanofibers were uniform and homogeneous. The sample deposited at 150 °C presented a beige color and the third sample, deposited at 150 °C followed by 250 °C, showed a yellowish color. Interestingly, the sample acquired a bluish hue when deposition occurred at 200 °C. The difference in coloration may be related to the optical interference owned to the number of cycles which can be occurred due to the superposition of the different layers of the deposited material [34]. In contrast, these results showed that not only the variation in the deposition cycles produced different colors but also a change in chamber’s temperature caused different tonalities.

**Figure 2:**
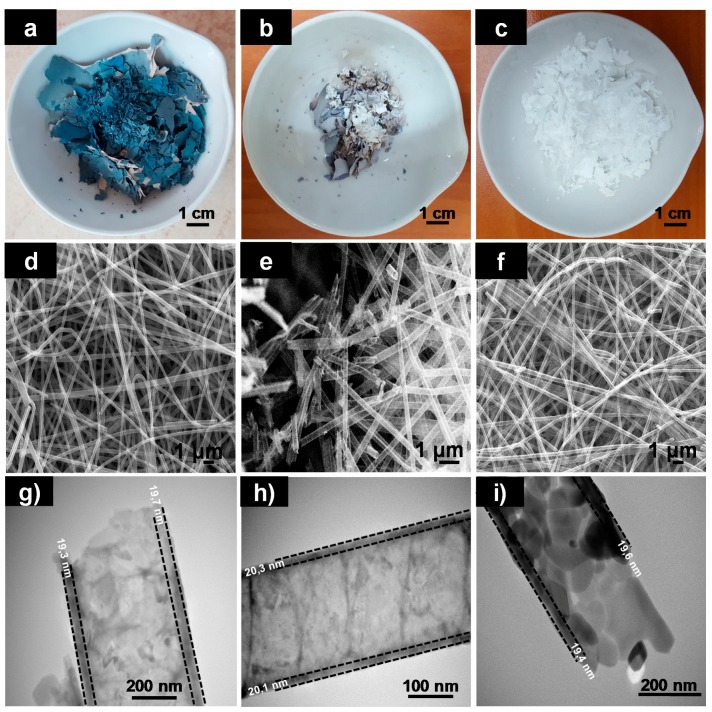
Photographs, SEM and TEM images of samples after removal processes. Photographs of: (**a**) TDN_A; (**b**) TDN_B400; (**c**) TDN_B600; SEM images of: (**d**) TDN_A; (**e**) TDN_B400; (**f**) TDN_B600; and TEM images of: (**g**) TDN_A; (**h**) TDN_B400 and (**i**) TDN_B600.

Subsequently, sample that presented highest deposition rate (PVN_200, according to thermal analysis in Section 3.2) suffered polymeric removal processes which were carried out with the purpose to obtain titanium dioxide nanotubes to enhance specific surface area of nanostructures. Figure 2 shows the color and the morphologies of titanium dioxides nanotubes (TDN) obtained using different methodologies aimed to remove PVN. In all cases, SEM and TEM images revealed that TiO_2_ deposition protected the morphology from removal processes and nanofibers served as an efficient “template” in the synthesis of titanium dioxide nanotubes.

The polymer removal process by washing maintained the initial color (sample TDN_A shown in Figure 2a). Unexpectedly, when the sample was heated at 400 °C, sample TDN_B400, the color of the nanotubes started to change from blue (Figure 1c) to white (Figure 2b), obtaining the total change to white at 600 °C (Figure 2c), sample TDN_B600. Certainly, this change of color was associated to TiO_2_ crystalline changes from amorphous phase to anatase crystalline structure (explained in next Section 3.4). Regarding to the thickness of TiO_2_ deposition, as Figure 2g–i shows, samples obtained after different polymer removal processes presented similar values. The results obtained after the measurement of 50 nanotubes were: (19.3 ± 2.7) nm, (20.1 ± 2.4) nm and (19.4 ± 2.6) nm for TDN_A, TDN_B400 and TDN_B600, respectively. TiO_2_ thickness did not present significant differences between samples, showing that deposition occurred at 200 °C exhibited high uniformity. Moreover, at this chamber temperature, highest ALD efficiency was achieved since Growth per Cycle (GPC) was approximately 0.04 Angstroms/cycle (in agreement with provider’s recipe).

On the other hand, nanotube diameters of samples with remaining PVN presented higher values than samples that suffered thermal decomposition at 600 °C. Probably, PVN suffered some swelling process due to the hydrophilic nature of this polymer and the presence of water as oxidant precursor during TiO_2_ ALD. And lately, nanotubes suffered some shrinking after thermal decomposition process, This fact was more accentuated at 600 °C [35].

**Figure 3:**
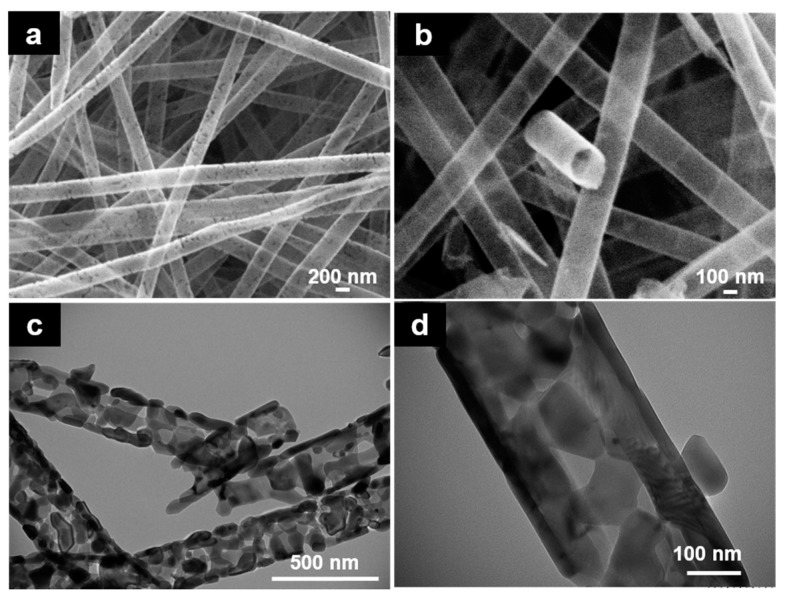
(**a**,**b**) SEM images of TDN_B600; (**c**,**d**) TEM images of TDN_B600 (20,000× and 60,000×, respectively).

In addition, SEM and TEM images of washed and thermal treated at 400 °C samples (Figure 2d,e,g,h) evidenced the presence of remainder polymer into the nanotubes and their morphologies did not suffer the same changes as sample TDN_B600 (Figure 2f,i) (more SEM and TEM images of TDN_A, TDN_400B and TDN_600B can be found in Supplementary Information, Figure S2). From now on, samples TDN_B600 will be named TDN since it was the selected methodology to remove polymer. SEM analysis confirmed that TDN were hollow and their walls have some porosities (Figure 3a,b).

The release of compounds resulted from the PVN degradation, such as water and acetaldehyde byproducts, during aggressive thermal treatment process (600 °C), could have generated sufficient pressure into the nanotubes resulting in these fractures in the nanotube walls. Figure 3c,d show the tubular structure of TDN was maintained with interconnected nanocrystal chains and their wall thicknesses were approximately 19–22 nm. Probably, these structures were produced by thermal exposure of titanium dioxide, which led to crystal phase change, from amorphous phase to anatase crystalline structure and to rutile at higher temperatures [23].

This small wall thickness is very interesting since other methods have resulted in nanoparticles with a larger particle size, such as the sol-gel method by Mosquera et al. that end up in particles with sizes between 114–171 nm [36]. Other works have shown higher metal oxide deposition rates, such as Park et al. studies where 500 cycles resulted in 80 nm zinc oxide depositions [37]. These results revealed that, in addition of temperature, the thickness of nanotubes resulting from ALD process is also clearly dependent on other factors, principally the substrate and the precursors used.

### 3.2. Thermal Characterization of Nanofibers and Nanotubes

**Figure 4:**
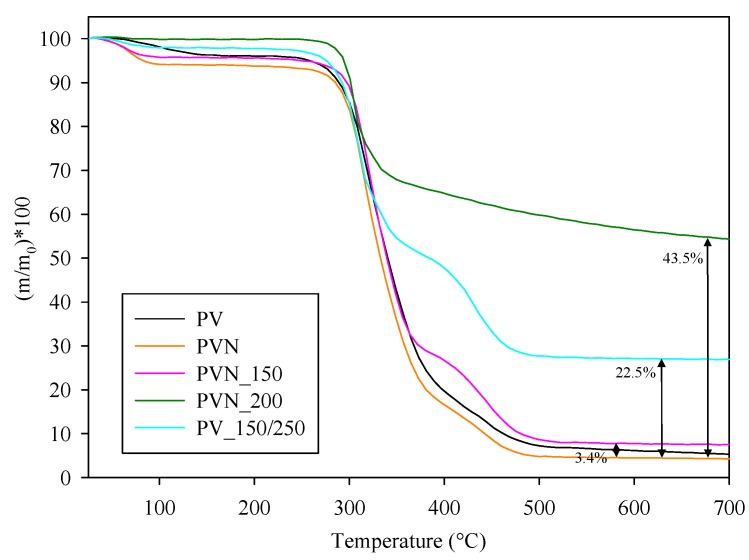
Weight loss with temperature of PVOH polymer (PV), electrospun nanofibers (PVN) and nanofibers coated at different temperatures with their corresponding %TiO_2_ deposited.

**Figure 5:**
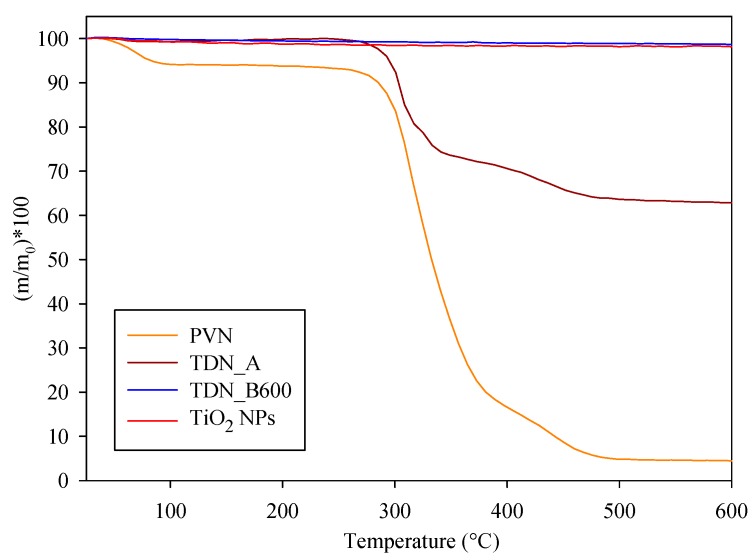
TGA curves of samples after polymer removal processes of TDN coated at 200 °C.

TGA curves of mass loss with temperature of PVN, PVN_TiO_2_ at different temperatures, TDN and commercial TiO_2_ NPs are presented in Figure 4 and Figure 5. TGA curves of PV and PVN presented common PV degradation processes: (i) below 100 °C, a 10% loss mass related with samples’ dehydration; (ii) a process between 220 °C and 390 °C related to the separation of side groups which formed water, acetic acid and acetaldehyde byproducts and the loss of hydrogen bond between PV chains and O–bond between C–O; and (iii) a process between 390 °C and 480 °C attributed to the degradation of principal chain of polymer [38,39]. In addition, PVN presented a maximum degradation temperature lower than PV polymer. This fact was already observed before and it is common in electrospun structures because a change occurred in the polymer structure to nanoscale during electrospinning process, increasing the specific surface and the heat penetrate faster [40].

Since titanium dioxide does not undergo degradation with temperature, the most important information obtained from these analyses was the quantification of TiO_2_ deposited over the nanofibers, which are shown in Figure 4 and expressed in %TiO_2_ respect total sample weight. ALD process at 150 °C (PVN_150) resulted in a low deposition rate but when the temperature of chamber increased to 200 °C (sample PVN_200) the percentage of titanium dioxide deposited achieved the highest value of 43.5%. Although other works have achieved high quality ALD depositions at low temperatures, ALD process parameters are highly dependent on ALD equipment. Some ALD processes have developed TiO_2_ thin films even at room temperature by using PE-ALD (ALD-enhanced plasma) or a home-made ALD with a very small camera [25,41]. On the other hand, Bishal et al. (2017) have recently evidenced that the deposition rate varied with gradually increasing temperature [25]. The third deposition (PVN_150/250) showed a titanium dioxide deposition value of 22.5%. The first 250 cycles at 150 °C were carried out in order to protect PVN structures to perform as template for a further deposition at 250 °C in case PV could be degraded at this temperature. Certainly, this low temperature was not sufficient to achieve efficient deposition with our equipment. According to these results, the deposition at 200 °C was the process selected to obtain the samples for further analysis.

TGA analysis was also useful to confirm the effectiveness of PV removal processes. Figure 5 revealed calcination at 600 °C was the unique process to produce the total PV removal. In addition, TDN and TiO_2_ samples showed a weight loss between 1–2%, which is related with the decomposition of same hydroxyls groups of titanium dioxide surface [42].

### 3.3. X-ray Analysis Results

**Figure 6:**
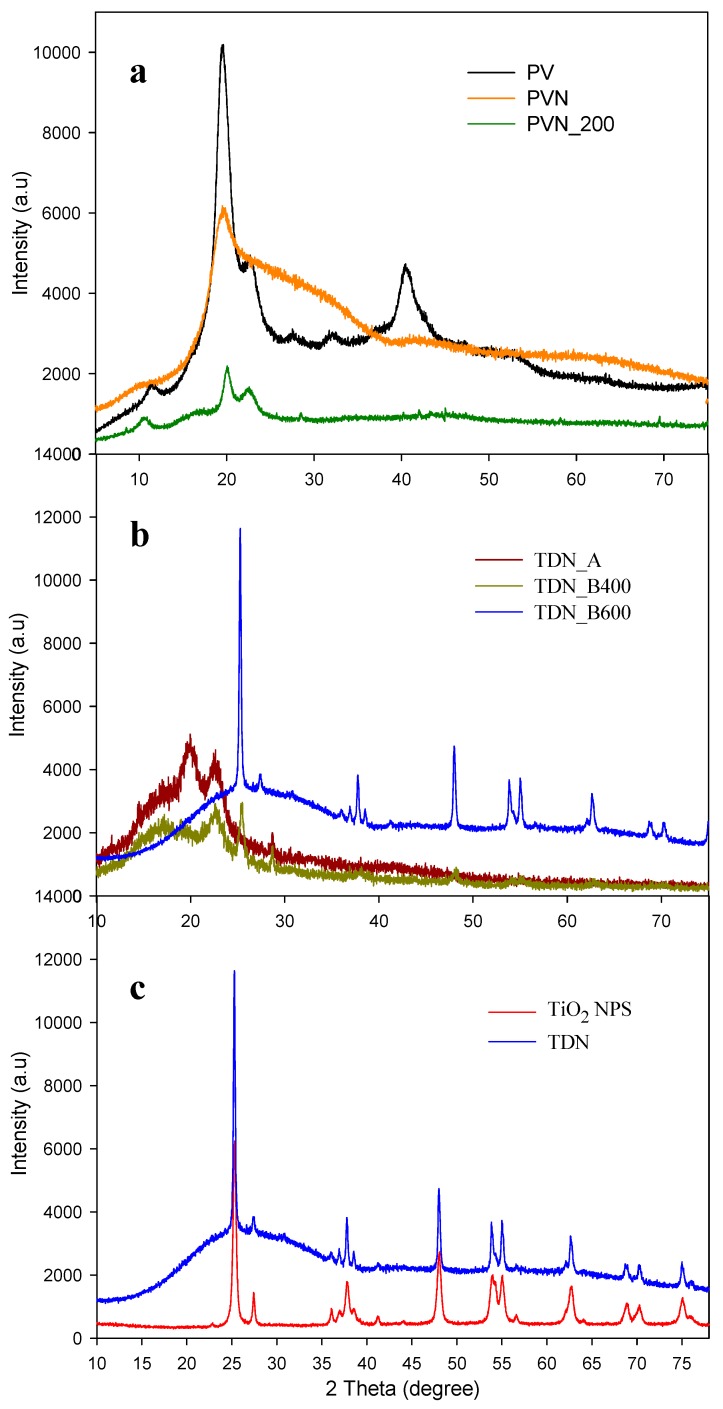
X-ray diffraction patterns of: (**a**) PV polymer and uncoated and coated through ALD PV electrospun nanofibers; (**b**) samples after removal polymer processes; and (**c**) commercial TiO_2_ NPs and titanium dioxide nanotubes (TDN).

XRD analyses of poly (vinyl alcohol) (PV), poly (vinyl alcohol) nanofibers (PVN) and deposited nanofibers at 200 °C by ALD (PVN_200) are plotted in Figure 6A. The PV diffraction pattern presented characteristic peaks at 2θ = 11.2, 19.5, 22.1, 32.1 and 40.2°, which were attributed to semi-crystalline nature of polymer [43,44,45]. On the other hand, the diffraction patterns of PVN presented an amorphous aspect, exhibiting only one peak at 2θ = 19.5°. Polymer crystallinity was highly affected by electrospinning process, where a fast speed of solvent evaporation led to a low molecular disposition [39,40]. The diffraction pattern of deposited PVN_200 presented some PV characteristic peaks at 2θ = 9.9, 19.9 and 22.0°. Likewise, a displacement of peak at 11.2° to 9.9° was observed. This fact was due to the deposition of titanium dioxide over the PVN. During ALD process, the precursors were deposited in the surface of PVN causing their coating. The displacement of the peak can be caused due to the intercalations between the deposited TiO_2_ and the PV polymeric chains. In addition, as Figure 6A shows, the deposited titanium dioxide presented an amorphous phase, because TiO_2_ layers did not present crystalline structure [34].

Figure 6B shows the effectiveness of the treatments to eliminate the polymer from nanotubes. PV residual in TiO_2_ nanotubes after washing and calcination at 400 °C was also confirmed by XRD analysis, showing an intense peak at 22.4°. In addition, the calcination at 400 °C showed other peak at 25.5° associated to the anatase crystalline structure of titanium dioxide. On the contrary, the calcination at highest temperature (600 °C) did not show any peak associated to PV polymer, only, a broad band between 15° and 35° was observed, which could be attributed to ash residues of polymer.

XRD diffraction patterns of commercial titanium dioxide (TiO_2_) nanoparticles and hollow titanium dioxide nanotubes (TDN corresponding to TDN_B600) presented several peaks, as shown in Figure 6C. Both TiO_2_ nanostructures evidenced characteristics peaks of anatase crystalline structure TiO_2_ at 2θ = 25.3, 36.8, 37.7, 38.1, 47.9, 53.8, 54.9, 62.6, 68.7, 70.1 and 74.9° [23,46,47]. In addition, peaks at 2θ = 27.4, 36.1 and 56.2° indicated that TiO_2_ also presented some nanoparticles with rutile crystalline structure [48,49]. Some studies have confirmed that anatase is a metastable structure which can be irreversibly transformed to an stable rutile structure between 400 °C and 1000 °C, but, in general this fact will be dependent of nanocrystal size, deviations of stoichiometry, superficial area, microstructure of anatase powder and temperature of thermal treatment [50,51]. Fortunately, this conversion to rutile did not occur, since it is more interesting to maintain anatase crystallographic structure due to their higher antimicrobial activity [52]. Likewise, the sizes of commercial TiO_2_ NPs and TDNs were calculated using Debye-Scherrer’s equation. Results indicated crystallite size of TDN was 25 nm and commercial TiO_2_ nanoparticles presented crystallite size of 20 nm, result that confirmed data sheet value.

### 3.4. FTIR Analysis Results

**Figure 7:**
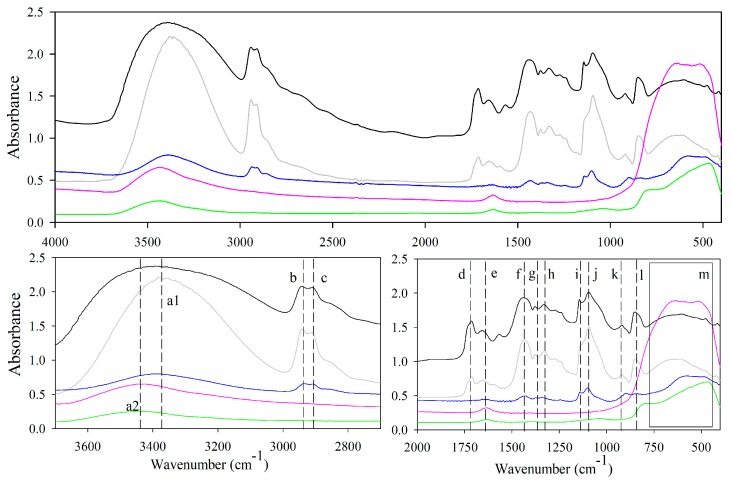
FTIR spectra of PV polymer (black), uncoated PVN (grey), ALD-coated PVN_200 (blue), commercial TiO_2_ NPs (pink) and TDN (green) and letter a-l correspond to specific peaks from Table 2.

Figure 7 shows FTIR spectra recorded of PV polymer, PVN and titanium dioxide nanostructures and their characteristic peaks are presented in Table 1. Although PV and PVN clearly presented the main absorption bands of this polymer, PVN nanofibers did not show the presence of the peak associated to the crystal sequence related to C–O stretching at 1143 cm^−1^ due to the reduction of crystallinity of the polymer caused by the electrospinning process, as it was already seen through DRX analysis [44,53,54]. This fact was also evidenced by a reduction of ratio (*D*_1145_/*D*_1096_) which had been closely related to degree of crystallinity [55].

FTIR spectra of commercial TiO_2_ NPs showed some characteristics peaks resulted from O–H interaction in the TiO_2_ surface (peak a2 at 3442 cm^−1^ in Figure 7) and the scissors type deformation of adsorbed water protons (peak e at 1637 cm^−1^) [36,50,54]. Although characteristic peaks of Ti–O bonds for anatase and rutile phases have been specifically identified by El-Sherbiny et al., other studies have also registered a very broad band in a range of 400–700 cm^−1^ due to the vibration of Ti–O–Ti bonds in TiO_2_ lattice [23,56,57,58,59]. PVN_200 spectra easily revealed the residual PV through the presence of PVN characteristics, such as the presence of peaks at 2941 cm^−1^, 2913 cm^−1^ and 1094 cm^−1^.

Additionally, a broad band appeared between 519 and 623 cm^−1^, which can be related to the stretching vibrations of Ti–O, Ti–O–C and Ti–O-Ti bonds [23,51]. Previous studies have confirmed bonding Ti–O–Ti bond appears when the TDMAT is introduced in the chamber and its molecules are chemisorbed by active sites of substrate surface, consuming –OH groups and producing reactive groups Ti–(N(CH_3_)_2_)_3_, which are present in the subsurface of substrate. Immediately, the purge with N_2_ eliminates byproducts and remaining TDMAT and the ultrapure water is introduced in the chamber, the oxidation process continues and Ti–O–Ti bonding is formed. Finally, a film of TiO_2_ is formed over the surface of substrate, where the titanium atoms are connected by oxygen atoms [34,51]. FTIR spectra of TDN showed similar peaks to TiO_2_ NPs associated to the stretching vibration of OH group (3443 cm^−1^), bending modes of water Ti–OH (1639 cm^−1^) and vibration of Ti–O–Ti bonds in the range between 400 and 800 cm^−1^ [23,58,60].

### 3.5. Antimicrobial Activities Results

Antimicrobial activities results are shown in Table 2 and were expressed as log reduction calculated following Equation (1). When exposed to UVA light, TDN presented interesting antimicrobial capacities and, as it was expected, the reduction in all bacterial concentrations increased as the concentration of titanium dioxide nanotubes increased. It is necessary to mention the effect of UVA irradiation on microorganisms was also checked and results verified that UVA irradiation inhibited bacteria negligibly, presenting only 0.1 log cycles reduction. TDN concentrations between 150 and 400 µg/mL resulted in high and sometimes total inhibition of studied bacteria.

When compared to other nanoparticles whose bactericidal properties were reported in previous works, TDN presented higher antibacterial capacities. Common nanoparticles concentrations used to inhibit Gram-positive and Gram-negative bacteria were markedly higher than TDN concentrations used in this work. Common applied dosages range of different nanoparticles, such as zinc oxide NPs, silver, nickel and copper oxide NPs were between 0.35 and 20 mg/L [61].

Due to the photocatalytic nature of titanium dioxide, one of the main mechanism of action of TDN is through the generation of reactive oxygen species (ROS) on its surface during the process of photocatalysis when they were exposed to light at an appropriate wavelength. The relationship between the absorption of energy by an electron to overcome from the valence gap to reach the conduction band, the generation of electron-hole pairs and the formation of different ROS and other radical species have been studied [62,63,64]. In addition, the destruction or damage of cell membrane as the main process for bacteria inactivation has been indirectly evidenced by studies of leakage of cellular components, such as potassium cations, RNA and protein [65,66].

In this study, antimicrobial activities of commercial TiO_2_ NPs were also evaluated in order to study the effect of morphology on antimicrobial ability (SEM image of TiO_2_ NPs can be found in Supplementary Information, Figure S3). As it can be observed in Table 2, both morphologies presented high antimicrobial activities but no trend was established since both nanostructures presented best performance against different microorganisms. TDN presented largest antimicrobial activity against *Escherichia coli*, presenting total bacteria reduction at 200 µg/mL. Antimicrobial effectiveness is strongly influenced by interaction between antimicrobial agent and microorganisms. Certainly, hollow TDN morphologies allowed a greater contact area (external and internal) with this bacterial solution, allowing a greater ROS formation. Furthermore, bactericidal action of nanoparticles typically involves a combination of physical and chemical mechanisms and many factors, such as size, shape and the nature of the target microorganism, influence on their toxicological profile toward microorganisms. As it was already commented with other antimicrobial nanotube structures, the mechanism of toxicity can be also influenced by other factors such as diameter, length and surface chemistry that influence on diffusion of bacteria into these structures. Research developed by Upadhyayula et al. to determine adsorption kinetics of *E. coli* and *S. aureus* on single-walled carbon nanotubes have revealed diffusivity of bacterial cells are concentration and bacteria dependent [67,68]. As a result, TDN antimicrobial activity was even higher than commercial TiO_2_ NPs. On the other hand, TDN antimicrobial power was lower against Gram-positive bacteria. Several studies have shown that Gram-positive bacteria were more resistant to photocatalytic disinfection than Gram-negative bacteria [69,70]. The main difference between both bacteria is the cell wall structure. Gram-negative bacteria have a triple-layer cell with an inner membrane, a thin peptidoglycan layer and an outer membrane, while Gram-positive bacteria have a thicker peptidoglycan layer and no outer membrane. Certainly, the thicker peptidoglycan layer implied a higher protection for Gram-positive bacteria.

TiO_2_ NPs showed highest antimicrobial activity against *S. aureus*. Probably, their size and morphology allowed them to have a better affinity with this bacterial cell, which produced greater contact and therefore higher log reduction efficiency. Ultimately, in the case of *L. innocua*, both TiO_2_ nanostructures presented similar activities and lowest antimicrobial values when compared with other bacteria. This high resistance by *Listeria* genera was already observed in other works [71].

The above results confirmed that antimicrobial activities are highly influenced by several factors including the nanostructures morphology, source, size, concentration and microorganism [9,10,13,72]. The control of morphology and nanostructure of TiO_2_ is an important factor to enhance its antimicrobial activity. In general, the appropriate design based on desirable surface properties given by shaped nanoparticles is dependent of type of bacteria. Other studies have already claimed the shape of the nanoparticles is one of the most important properties which affects their physico-chemical properties [73].

## 4. Conclusions

In summary, this research has shown the combination of electrospinning and atomic layer deposition techniques as an attractive way to obtain novel metal oxide nanostructures with promising antimicrobial purposes. Titanium dioxide nanotubes were satisfactorily developed through atomic layer deposition of tetrakis (dimethylamide) titanium (TDMAT) and ultra-pure water, as precursors, over polyvinyl alcohol electrospun nanofibers. Thermal and structural properties studied through thermogravimetric, XRD, FTIR, SEM and TEM analysis were of great importance to understand the process during all stages. The highest TiO_2_ deposition efficiency was achieved with an ALD temperature chamber of 200 °C and thermal treatment of deposited samples at 600 °C was the most efficient process to remove PV polymer in order to obtain hollow TiO_2_ nanotubes (TDN). The specific mechanisms that govern the antimicrobial activities of TDN was not studied in this research and, as with other nanoparticles, it is hugely challenging to understand individual and synergistic contributions of physical, chemical and electrical effects of nanoparticles on cells. Antimicrobial analysis revealed TDN showed higher bactericidal power against *Escherichia coli* than commercial TiO_2_ nanoparticles but lower activity in the case of *Staphylococcus aureus*. Results evidenced bactericidal activities are highly dependent of many factors, including intrinsic properties of nanomaterials and bacteria type.

## Figures and Tables

**Table 1:** Characteristic wavenumbers expressed in (cm^−1^) associated to assignments of FTIR absorption bands of PV polymer, PVN and TiO_2_ nanostructures.

Peaks	PV Polymer	PVN	TiO_2_ NPs	TDN	Assignment
a	3384(a1)	3380(a1)	3442(a2)	3443(a2)	O–H stretching
b	2942	2941	-	-	–CH_2_– stretching
c	2910	2913	-	-	–CH_2_– symmetrical and asymmetrical stretching
d	1715	1714	-	-	C=O, C–O band from carbonyl group
e	-	-	1637	1639	bending modes of water Ti–OH
f	1435	1434	-	-	CH_2_, O–H and C–H bending
g	1377	1377	-	-	CH_2_ wagging
h	1333	1330	-	-	O–H in-plane bending with C–H wagging
i	1143	-	-	-	C–C stretching, O–H bending, C–O–C, C–O
j	1094	1094			CO stretching, OCC antisymmetric stretching
k	919	920	-	-	CH_2_ bending
l	851	849	-	-	CH_2_ rocking
m	-	-	700–400	800–400	Ti–O–Ti bonding

**Table 2:** Antimicrobial results of TDN and commercial TiO_2_ NPs at different concentrations against different microorganisms.

Microorganism:	*Escherichia coli*		*Staphylococcus aureus*		*Listeria innocua*	
**TDN (µg/mL)**	**Cel. conc. (cfu/mL)**	**Log Reduction**	**Cel. conc. (cfu /mL)**	**Log Reduction**	**Cel. conc. (cfu /mL)**	**Log Reduction**
0	(3.95 ± 0.16) × 10^5^	0 ^a^	(2.02 ± 0.12) × 10^5^	0 ^a^	(5.93 ± 0.38) × 10^5^	0 ^a^
100	(5.36 ± 0.29) × 10^4^	0.87 ± 0.02 ^b^	(3.14 ± 0.34) × 10^4^	0.81 ± 0.04 ^b^	(1.01 ± 0.63) × 10^5^	0.84 ± 0.26 ^b^
150	(8.67 ± 0.47) × 10^2^	2.66 ± 0.03 ^e^	(1.27 ± 0.28) × 10^4^	1.21 ± 0.08 ^c^	(4.46 ± 0.25) × 10^4^	1.12 ± 0.02 ^c^
200	0	5.59 ^g^	(1.32 ± 0.22) × 10^3^	2.19 ± 0.06 ^d^	(4.20 ± 0.27) × 10^4^	1.15 ± 0.02 ^c,d^
400	0	5.59 ^g^	(5.33 ± 0.23) × 10^2^	2.58 ± 0.02 ^e^	(5.05 ± 0.07) × 10^3^	2.07 ± 0.01 ^f^
**TiO_2_ NPs (µg/mL)**	**Cel. conc. (cfu /mL)**	**Log Reduction**	**Cel. conc. (cfu /mL)**	**Log Reduction**	**Cel. conc. (cfu /mL)**	**Log Reduction**
0	(4.58 ± 1.52) × 10^5^	0 ^a^	(1.98 ± 0.29) × 10^5^	0 ^a^	(3.57 ± 0.48) × 10^5^	0 ^a^
100	(5.42 ± 0.45) × 10^3^	1.93 ± 0.04 ^c^	(2.75 ± 0.19) × 10^2^	2.86 ± 0.02 ^f^	(1.42 ± 0.21) × 10^4^	1.40 ± 0.05 ^e^
150	(2.77 ± 0.48) × 10^3^	2.22 ± 0.08 ^d^	(1.67 ± 0.89) × 10^2^	3.12 ± 0.21 ^g^	(1.63 ± 0.65) × 10^4^	1.37 ± 0.15 ^d,e^
200	(5.17 ± 0.88) × 10^2^	2.95 ± 0.07 ^f^	0	5.36 ^h^	(1.03 ± 0.12) × 10^3^	2.54 ± 0.04 ^g^
400	0	5.66 ^g^	0	5.36 ^h^	(7.17 ± 0.95) × 10^2^	2.69 ± 0.05 ^g^

^a–h^ Means values within a column with different superscripts differ significantly (*p* < 0.05).

## Supplementary Materials

The following are available online at http://www.mdpi.com/2079-4991/8/2/128/s1. Contents: Section S1.1. Study of the effect of electrospinning parameters on fiber diameter; Section S1.2. Provider’s recipe Cambridge Nanotech; Section S2.1. Morphological results of nanofiber; Section S2.2. Morphological results of TiO_2_ nanostructures.
