# Supplementary material for: Novel Antimicrobial Titanium Dioxide Nanotubes Obtained through a Combination of Atomic Layer Deposition and Electrospinning Technologies
Source: Nanomaterials (Basel). 2018 Feb 24;8(2):128. doi: 10.3390/nano8020128 (PMC5853759; doi:10.3390/nano8020128)
Supplement: Supplementary file 1 [file nanomaterials-08-00128-s001.pdf]

Supporting Information

2SI. Experimental Section

2S.1. Study of the effect of electrospinning parameters on fiber diameter

Poly (vinyl alcohol) nanofibers (PVN) were obtained with a polymeric solution at 8% (*w*/*w*) using an electrospinning system (Spraybase® power Supply Unit, Ireland). 1.6 g poly (vinyl alcohol) (PV) was added to 20 mL of distilled water and stirred at 90 °C until polymer was dissolved. Solution were transferred to 5 mL plastic syringes and connected through a PTFE tube to a stainless steel needle charged by a high voltage power supply with a range of 0-20 kV. The parameters of electrospinning system such as distance (height between tip of the needle and collector plate), diameter of needle and flow rate were studied in order to determine their effect on the PVN diameter. The variables studied were: distance (8.5, 10 and 12 cm), diameter of needle (0.45, 0.7, 0.9, 1.2, 1.6 mm) and flow rate (0.75, 1.0 and 1.5 mL/h). Fiber diameter was measured using program Image J.

2S.2. Provider´s recipe Cambridge NanoTech

Figure 1SI presents the Cambridge NanoTech´s recipe in order to observe the different purge times used for different temperatures on atomic layer deposition of Tetrakis(dimethylamido)titanium (TDMAT). These instructions were followed during this work.

CambridgeNanoTech
Simply ALD

TiO_2 – Titanium Dioxide

Savannah Film Recipe

Precursrors:

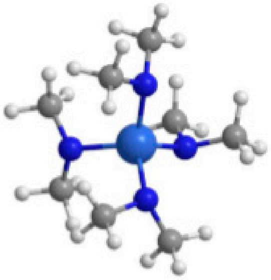

Tetrakis(dimethylamido)titanium, TDMAT, $Ti(NMe_2)_4$
CAS#: 3275-24-9
Heated to 75°C

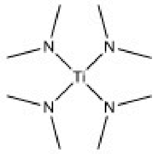

H_2O, unheated

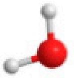

250°C growth:
8=9=250°C, 6=7=10=150°C, flow=20sccm

pulse $Ti(NMe_2)_4$ (Valve 1) , 0.1 pulse
wait 5s
pulse H_2O (Valve 0) , 0.015sec pulse
wait 5s

Growth per cycle: ~0.39Å/cycle
Refractive Index: between 2.1-2.5 depending on film thickness

	Instruction	#	Value
0	flow		20
1	heater	9	250
2	heater	8	250
3	stabilize	9	
4	stabilize	8	
5	wait		600
6	pulse	1	0.10
7	wait		5
8	pulse	0	0.015
9	wait		5
10	goto	6	m
11	flow		5

200°C growth:
8=9=200°C, 6=7=10=150°C, flow=20sccm

pulse $Ti(NMe_2)_4$ (Valve 1) , 0.1 pulse
wait 10s
pulse H_2O (Valve 0) , 0.015sec pulse
wait 10s

Growth per cycle: ~0.40Å/cycle
Refractive Index: between 2.2-2.5 depending on film thickness

	Instruction	#	Value
0	flow		20
1	heater	9	200
2	heater	8	200
3	stabilize	9	
4	stabilize	8	
5	wait		600
6	pulse	1	0.10
7	wait		10
8	pulse	0	0.015
9	wait		10
10	goto	6	m
11	flow		5

R090111

One Kendall Square, Suite B7301
Cambridge, MA 02139, USA

T: 617-674-8800
F: 617-674-8850
www.cambridgenanotech.com

TiO$_2$ (TDMAT) Savannah Recipe – Page 2

CambridgeNanoTech
Simply ALD

150°C growth:
8=9=150°C, 6=7=10=150°C, flow=20sccm

pulse Ti(NMe$_2$)$_4$ (Valve 1) , 0.1 pulse
wait 20s
pulse H_2O (Valve 0) , 0.015sec pulse
wait 20s

Growth per cycle: ~0.40Å/cycle
Refractive Index: between 2.2-2.5 depending on film thickness

	Instruction	#	Value
0	flow		20
1	heater	9	150
2	heater	8	150
3	stabilize	9	
4	stabilize	8	
5	wait		600
6	pulse	1	0.10
7	wait		20
8	pulse	0	0.015
9	wait		20
10	goto	6	m
11	flow		5

Application Notes:

ALD TiO_2 can also be deposited from titanium(IV) tetraisoproxide, Ti(OiPr)$_4$, titanium(IV) methoxide, Ti(OCH$_3$)$_4$, titanium(IV) ethoxide, Ti(OC$_2$H$_5$)$_4$ or titanium tetrachloride, $TiCl_4$. Titantium(IV) tetraisoproxide has a short lifetime in the precursor cylinder when kept at volatilization temperature. Titanium(IV) methoxide and titanium(IV) ethoxide are low volatility solids which are suitable for high temperature TiO_2 processes (>250°C) and require advanced volatilization techniques for introduction into the system (ALD Boost). Titanium tetrachloride, $TiCl_4$, is not recommended as the byproduct of the reaction is HCl which can etch the growing film and contaminate the reactor with chlorine.

With a deposition temperature below ~180°C, as-deposited TiO_2 films are amorphous. At temperatures higher than 180°C, TiO_2 films will contain increasing amounts of anatase as the deposition temperature rises. If annealed at temperatures above 400°C, rutile TiO_2 will be formed.

TiO_2 depositions become substrate inhibited or the growth-per cycle drops after ~350Å of film deposition due to electronic and/or hydroxyl effects of the film.

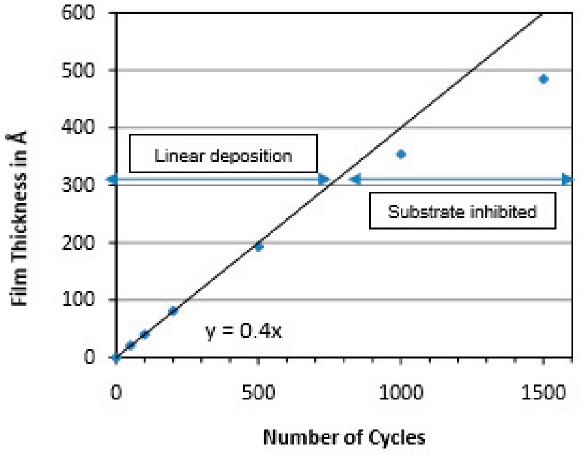

Fig. 1SI. Provider´s recipe of ALD of TMDA. **3SI. Results and discussion**

3S.1 Morphological Results of Nanofiber

Electrospun PVN were successfully obtained with a polymeric solution at 8% (*w*/*w*). This concentration was fixed because is one the most important parameters to obtain uniform nanofibers and maintain a stable electrospinning process.[48] Earlier experiments have shown that the use of polymeric solutions at lower concentrations produced flow instability and presence of drops, while the performance using high concentrations produced changes in the nanofiber morphology. The effect of the needle diameter, the flow rate and the distance between the needle and the collector on the diameter of nanofibers was studied in order to obtain the nanofibers with lowest diameter. All experiments resulted on thin, circular and uniforms PVN, but the nanofiber with lowest diameter was obtained using a needle diameter, 10 cm and flow rate 1.75 mL/h. The diameters of nanofibers obtained are presented in Table 1SI**,** and all experiments resulted on thin, circular and uniform PVN, as Fig. 2SI shows. The diameter of nanofibers slightly increased when the needle diameter increased from 0.45 to 0.90 mm, but those values significantly decreased using higher values, obtaining the lowest value of fiber diameter when needle diameter was 1.60 mm. The diameter of the needle influences greatly on the formation and stability of a stable Taylor's cone and the polymer stretching, and in this case, the lowest nanofiber diameter was obtained with the highest diameter of needle. In addition, the morphologies of nanofibers changed of wide to thin, as indicate Fig. 2SIa–e.

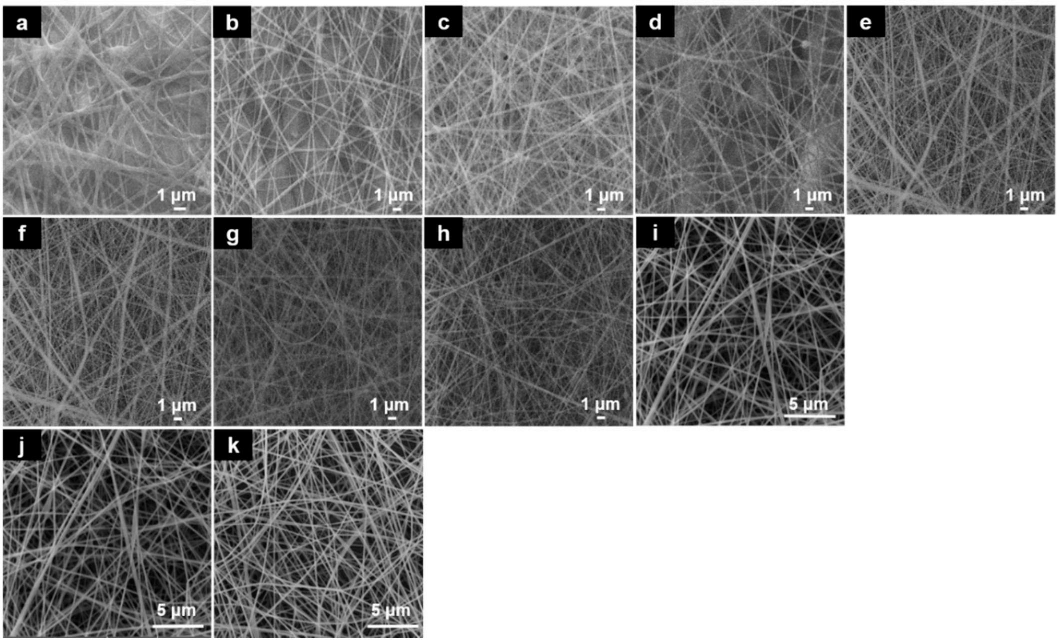

Fig. 2SI. SEM images of PVN according to conditions expressed in Table 1SI: (a–e) variation of needle diameter; (f–i) variation of flow rate; and (j-k) variation of the distance. All samples presented magnification 25000x.

Generally, the variation in the flow rate doesn't produce significant differences between the diameters of PVN, although the voltage applied increased (data not shown). When the flow rate increased, a high

electrical field was necessary to reach an increase in the coulombic stretching forces and electrostatic forces to produce thin nanofibers. By last, when needle diameter and flow rate were fixed, the effect of distance between needle and collector were studied and it was observed that the diameter of nanofibers decreased significantly until approximately 164.5 nm using a distance of 10 cm. At higher distance values, the presence of drops and unstable Taylor's cone did not allow electrospinning process.

Table 1SI. Diameters of nanofibers obtained.

Needle diameter (mm) / Sample	0.45 / A	0.70 / B	0.90 / C	1.20 / D	1.60 / E
Diameter (nm)	194.9 ± 36.3[a]	220.0 ± 36.8[b]	236.3 ± 33.2[b]	219.1 ± 24.7[b]	187.7 ± 23.8[a]
Flow rate (mL/h) / Sample	0.75 / F	1.00 / G	1.50 / H	1.75 / I	
Diameter (nm)	187.7 ± 23.8[bc]	192.9 ± 30.2[c]	170.9 ± 25.8[a]	177.9 ± 23.3[ab]	
Distance (cm) / Sample	8.5 / J	10 / K	12 / L		
Diameter (nm)	177.9 ± 23.3[b]	164.5 ± 24.7[a]	-		

Lower case letters a–c indicate significant differences among the parameters analyzed.

3S.2 Morphological Results of TiO_2 Nanostructures

Figure 3SI presents extra SEM and TEM images of TiO_2 nanotubes obtained after different polymeric removal processes.

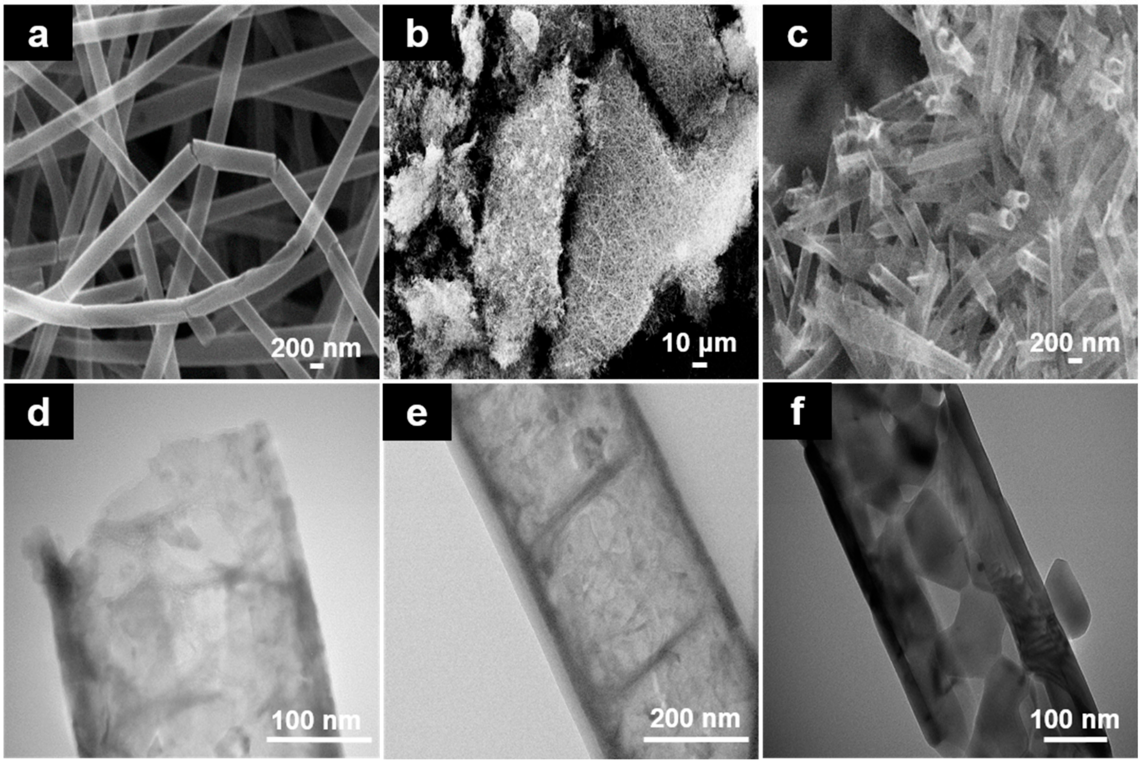

Figure 3SI. SEM images of: (a) TDN_A; (b) TDN_B400; (c) TDN_B600; and TEM images of: (d) TDN_A; (e) TDN_B400 and (f) TDN_B600.

On the other hand, Fig. 4SI presents TEM images of TiO_2 NPs in order to show different morphology of these nanostructures. TiO_2 NPs presented some spherical shape with a size average of approximately (22.9 ± 5.7) nm. Results revealed commercial nanoparticles presented higher deviation on sizes than deposited diameters of developed nanotubes.

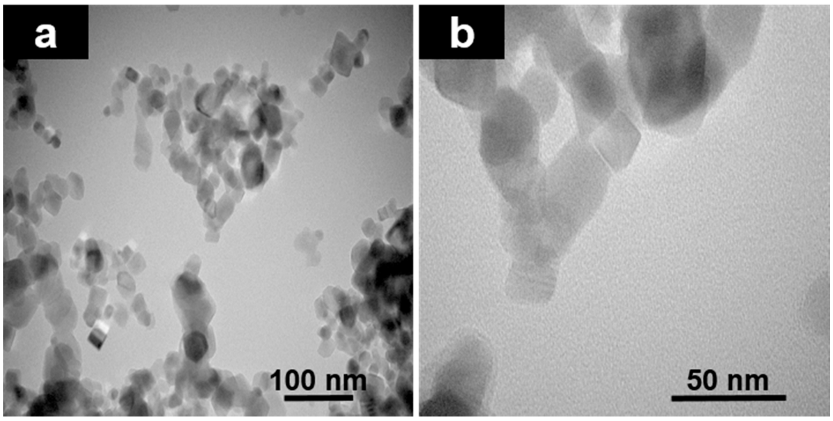

Fig. 4SI. SEM images of TiO_2 NPs at different magnifications: (a) 60 k× and (b) 200 k×.
